# Explainable Artificial Intelligence in Quantifying Breast Cancer Factors: Saudi Arabia Context

**DOI:** 10.3390/healthcare12101025

**Published:** 2024-05-15

**Authors:** Turki Alelyani, Maha M. Alshammari, Afnan Almuhanna, Onur Asan

**Affiliations:** 1 Department of Information Systems, College of Computer Science and Information Systems, Najran University, Najran 1988, Saudi Arabia; 2 Department of Environmental Health, Institute for Research and Medical Consultations, Imam Abdulrahman Bin Faisal University, Dammam 31441, Saudi Arabia; mmashammari@iau.edu.sa; 3 Department of Radiology, College of Medicine, Imam Abdulrahman Bin Faisal University, Dammam 31441, Saudi Arabia; amuhanna@iau.edu.sa; 4 School of Systems and Enterprises, Stevens Institute of Technology, Hoboken, NJ 07030, USA; oasan@stevens.edu

**Keywords:** artificial intelligence, machine learning, explainable artificial intelligence, breast cancer, classification, Saudi Arabia

## Abstract

Breast cancer represents a significant health concern, particularly in Saudi Arabia, where it ranks as the most prevalent cancer type among women. This study focuses on leveraging eXplainable Artificial Intelligence (XAI) techniques to predict benign and malignant breast cancer cases using various clinical and pathological features specific to Saudi Arabian patients. Six distinct models were trained and evaluated based on common performance metrics such as accuracy, precision, recall, F1 score, and AUC-ROC score. To enhance interpretability, Local Interpretable Model-Agnostic Explanations (LIME) and SHapley Additive exPlanations (SHAP) were applied. The analysis identified the Random Forest model as the top performer, achieving an accuracy of 0.72, along with robust precision, recall, F1 score, and AUC-ROC score values. Conversely, the Support Vector Machine model exhibited the poorest performance metrics, indicating its limited predictive capability. Notably, the XAI approaches unveiled variations in the feature importance rankings across models, underscoring the need for further investigation. These findings offer valuable insights into breast cancer diagnosis and machine learning interpretation, aiding healthcare providers in understanding and potentially integrating such technologies into clinical practices.

## 1. Introduction

Over the past decade, there has been a significant growth in cancer diseases in the Kingdom of Saudi Arabia. Several types of cancer, including colorectal, NHL, leukemia, prostate, lung, liver, Hodgkin’s lymphoma, thyroid, kidney, brain, and breast, have been increasing since 2012 [1]. For example, in 2020, the estimated mortality rate for this disease reached 685,000, accounting for approximately 13.6% of all female cancer-related deaths [2]. In the Kingdom of Saudi Arabia, breast cancer constitutes approximately 15.9% of the diagnosed cancer cases, as reported by the Ministry of Health in 2014 [3]. According to the International Agency for Research on Cancer in 2020 [4], 27,885 new cancer cases and 13,069 deaths were identified in 2020 in Saudi Arabia. Colorectal cancer was identified as having a high prevalence (14.4%), followed by breast cancer (14.2%), when both genders (13,632 females and 14,253 males) of all age groups were considered. Colorectal cancer was identified to be prevalent among males (19.3%), and breast cancer was identified to be prevalent among females (29%). A total of 82,640 cases (39,241 males and 43,399 females) were identified over the past five years, out of which breast cancer ranks first in relation to new cases, with a cumulative risk of 3.01, and ranks second in relation to deaths over the past few years when all types of cancer are considered, with a cumulative risk of 0.93. The effective management of cancer diseases in Saudi Arabia has been hampered by several notable challenges. Among these challenges are the limited availability of early detection screening programs, insufficient management guidelines, and a scarcity of resources for diagnosis and treatment [5].

Among the various types of cancers prevailing in Saudi Arabia, breast cancer is one of the major cancer types causing concern among younger females compared to Western countries [6]. Several factors have been associated with the cause and determinants or predictors of breast cancer in Saudi Arabia. These include factors such as age at first birth, early menarche, gender, dietary factors, tobacco smoking, alcohol consumption, low-dose irradiation, obesity, physical activity, lactation, hormonal factors, hormone replacement therapy, steroid hormone receptors, mammographic density, benign breast disease, and genetic factors [6]. Given the rising incident rates of breast cancer and a wider range of etiological factors, there is an immediate need to adopt preventive strategies.

Artificial intelligence (AI) has proven its extraordinary abilities in solving a wide range of tasks, resulting in widespread adoption in several industries. However, AI-based solutions in healthcare have often been met with skepticism among practitioners. This is due to the fact that these solutions operate using internal reasoning that is not transparent, and their decisions are difficult to explain, causing healthcare professionals to have reservations in trusting and comprehending them [7]. To address this issue, a new field within AI called eXplainable AI (XAI) is gaining attention from both academic and industrial researchers. In this research, our primary objective is to leverage the power of XAI to facilitate the early and transparent detection of breast cancer. Early detection can lead to timely treatment, improved prognosis, and significantly increase survival rates. Additionally, the accurate classification of symptoms and tumors can help patients avoid unnecessary treatments [8]. Machine Learning (ML) techniques have demonstrated their robustness in the development of detection algorithms [9]. In this study, we aim to explore a breast cancer dataset sourced from the University’s King Fahd Hospital (KFUH), in Dammam, Kingdom of Saudi Arabia, using ML techniques. The dataset comprises a range of features extracted from patients’ medical history, accompanied by corresponding diagnostic labels indicating whether it is malignant or benign.

## 2. Background

Machine learning improves prediction accuracy without explicit programming and has diverse applications. In breast cancer, ML techniques positively impact diagnosis and survival prediction by analyzing imaging and blood sample data, and optimize treatments. However, there is a scarcity of ML research on breast cancer in Saudi Arabia. Interpretability techniques like LIME and SHAP address the transparency and trust issues in ML models. Table 1 showcases research studies utilizing ML techniques in breast cancer diagnosis, demonstrating their application in classifying various types of data, including tumor symptoms, blood sample indicators, and imaging data.

### 2.1. Machine Learning for Accurate Predictive Analytics

ML, a subset of Artificial Intelligence, enhances the accuracy of prediction without explicit programming [25]. It finds applications in email filtering, threat detection, the automation of business processes, and areas requiring accurate predictive analytics [26]. ML encompasses supervised, unsupervised, semi-supervised, and reinforcement learning. Supervised learning employs training data with defined variables [27,28], while unsupervised learning identifies connections in unlabeled data [29]. Semi-supervised learning allows the autonomous exploration of training data [30], and reinforcement learning enables algorithmic decision making in multi-step processes [31]. ML utilizes various models trained on sample data, including Artificial Neural Networks (ANNs), Bayesian networks, and Support Vector Machines (SVMs) [32,33,34].

### 2.2. Machine Learning Techniques for Breast Cancer

ML techniques have been extensively studied and shown positive impacts in various areas, including the classification of diagnostic data, the prediction of survival, and assessing the risk of developing breast cancer [35,36,37,38,39,40]. Tahmassebi et al. [40] evaluated the impact of ML using multiparametric MRI (mpMRI) for early breast cancer prediction and survival outcomes. They identified relevant features for disease-specific survival prediction, such as the lesion size, volume distribution, and mean plasma flow. Similarly, Sultan et al. [41] employed ML algorithms on grayscale and Doppler ultrasound images of breast lesions to diagnose breast cancer with higher accuracy. In another study, Kate and Nadig [42] utilized ML models to predict breast cancer survivability at different stages of the disease. They found that the models achieved accurate predictions at each stage, but the training did not help when examples from other stages were used.

ML techniques have also been applied to analyze data from blood samples. Aslan et al. [37] employed four different techniques, including ANN, SVM, K-NN, and extreme learning machine (ELM), on a clinical dataset of blood samples. The dataset included parameters such as glucose, insulin, HOMA, leptin, adiponectin, resistin, and MCP1. They observed that ELM outperformed ANN with an accuracy of 83.8% compared to 77% for ANN.

Furthermore, ML techniques have been used to predict the need for different types of treatments, such as surgery, and to avoid unnecessary procedures. Bahl et al. [43] examined high-risk breast lesions (HRLs) diagnosed with image-guided needle biopsy. In their study analyzing 1006 HRLs, they found that 97.4% (37 out of 38) of malignancies could have been diagnosed through surgery, while 30.6% (91 out of 297) of surgeries for benign lesions could have been avoided. These studies demonstrate the effectiveness of ML techniques in breast cancer diagnosis, survival prediction, analyzing imaging and blood sample data, and optimizing treatment decisions.

### 2.3. Scarcity of Machine Learning Research in Saudi Breast Cancer

The extensive literature review conducted in this study identified that there are very few studies focusing on research related to the application of ML and AI techniques in breast cancer diagnosis and screening in Saudi Arabia. Zain et al. [44,45,46] studied the effectiveness of various ML algorithms including NB, K-NN, and fast decision tree (REPTree) for predicting breast cancer recurrence and found that K-NN produced a better prediction without principal component analysis (F-measure = 72.1%). Similarly, Sultana et al. [47], using SVM and Multi-Classifiers, identified that SVM offered a high accuracy and F-score in comparison with multi-classifiers. El Rahman [48], in a different context, proposed an algorithm for improving the methods used to detect breast cancer by analyzing DNA data and detecting the problem in DNA samples in Saudi Arabia. The need for extensive research in this field was considered to be of high importance, given the rising number of new cases related to breast cancer in Saudi Arabia, which is actively deploying innovative technologies for healthcare services as a part of its transformation from a traditional to digital and sustainable healthcare system [49].

### 2.4. Machine Learning Interpretation and Explanation in Breast Cancer

The field of artificial intelligence (AI) has witnessed significant advancements, particularly in machine learning (ML) algorithms. While these algorithms can achieve impressive results in various tasks, their internal workings are often complex and opaque [31]. This lack of transparency can hinder trust and limit the practical application of ML models, especially in healthcare settings where interpretability and the justification of decisions are crucial. Explainable Artificial Intelligence (XAI) addresses this challenge by encompassing a set of techniques that aim to make the internal workings and decision-making processes of ML models interpretable by humans [31]. By incorporating XAI methods, we can gain insights into how models arrive at their predictions, fostering trust and enabling a deeper understanding of the factors influencing the model’s outputs.

In the field of breast cancer detection and treatment, various models in deep learning and machine learning, such as gradient boosting and random forest algorithms, have been utilized [32,33]. To address the need for interpretability in the healthcare industry, research has focused on overcoming the transparency issues associated with machine learning models. One approach is LIME (Local Interpretable Model-Agnostic Explanations), which has been introduced to resolve transparency and trust issues in AI model predictions [50]. LIME is an interpretable model that plays a crucial role in building trust in the field of artificial intelligence and machine learning. It ensures that the classifiers in LIME make predictions in an uninterrupted manner, helping to maintain trust in the outputs. By addressing the lack of clarity faced by doctors during breast cancer detection, LIME contributes to making the diagnostic process more transparent and understandable [50].

In the field of machine learning, understanding intractability is crucial. Doshi [34] employed interpretability tools, such as Generalized Additive Models (GAMs) and the SHAP Python package, to examine their functionality and identify potential issues. Bua [35] introduced the network dissection framework to quantify latency-causing representations in convolutional neural networks, highlighting the synchronization between hidden units and semantic concepts. They resolved latency issues using supervised and unsupervised learning techniques.

SHAP (Shapley additive explanations), introduced by Lundberg and Lee in 2017 [36], addresses the challenge of interpreting machine learning results accurately. It assigns a unique identifier to each feature and consists of two components: measuring the importance of predictions based on additive features and providing theoretical results with appropriate solutions. Although SHAP has resolved many issues, it is not recommended for deep learning algorithms due to its slow performance. EL5 (Explain Like I’m 5) [38] follows the principles of LIME and aims to address questions at the level of a five-year-old child. It utilizes datasets, web-based articles, PDFs, and documents to provide comprehensive answers to simple questions.

### 2.5. Research Objective

The main objective of our research, using eXplainable Artificial Intelligence (XAI), is to identify the key risk factors associated with breast cancer in the Saudi Arabian population and quantitatively analyze the degree of their influence using machine learning. We leverage eXplainable Artificial Intelligence (XAI) techniques to enhance the interpretability of our machine learning models when used for breast cancer prognosis. By incorporating XAI, we aim to improve trust and transparency in the model’s decision-making process. Understanding how the model arrives at its predictions can empower healthcare professionals to have greater confidence in its outputs and facilitate clear communication with patients.

Through the use of XAI on a real dataset, we identified several crucial risk factors, including age, gender, age at menarche, age of first birth, family history, hormone replacement therapy, and breast density. Our research problem centered around determining which factors might indicate a higher risk of breast cancer and analyzing the degree of their effect. By doing so, we hope to provide valuable insights that healthcare providers can use to establish effective screening and early detection protocols. In our research, we endeavor to address the following questions:

RQ1: Do the proposed ML models generate consistent lists of the most important features?

RQ2: Which features have the most significant impact on the model’s decision-making process?

RQ3: What obstacles are encountered in the context of our study when implementing eXplainable Artificial Intelligence (XAI)?

To the best of the authors’ knowledge, our study is the first of its kind to investigate Saudi Arabian data in a manner similar to ours. This uniqueness highlights the significance of our research, as it fills a gap in the existing knowledge. The outcomes of our research have the potential to inform decision making, shape policies, and guide future studies, benefiting stakeholders in Saudi Arabia and beyond.

## 3. Materials and Methods

### 3.1. Data Source

We obtained our dataset from the University’s King Fahd Hospital (KFUH), affiliated with Imam Abdulrahman Bin Faisal University (IAU) in Dammam, Kingdom of Saudi Arabia. Our study was conducted with the approval of the Standing Committee for Research Ethics on Living Creatures (SCRELC), and we received ethical approval with reference number IRB -2020-13-371. The dataset consists of 4206 cases reported between 2017 and 2018, each with a patient ID number and a malignant or benign breast cancer diagnosis. We extracted several clinical and demographic features, including age, gender, age at menarche, age at first birth, family history, hormone replacement therapy, and breast density. Table 2 provides basic descriptive statistics for these features.

### 3.2. Machine Learning for Diagnosis Prediction

We investigated how machine learning can predict patient diagnoses using various algorithms. Each model’s hyperparameters were carefully tuned to optimize the performance for Bi-RADS score prediction. Here is an overview of the models used:

1. Random Forest: Tuned parameters include the number of trees, their maximum depth, and the minimum samples per leaf. This helps prevent overfitting and improves generalization to unseen data.
2. Logistic Regression: Regularization strength (C) was tuned to control the model’s complexity and prevent overfitting, leading to more accurate predictions.
3. Decision Trees: Tuning focused on the maximum depth, minimum samples per leaf, and splitting criteria (like Gini impurity) to control the tree’s complexity and enhance interpretability and generalizability.
4. Neural Network: The number of hidden layers, neurons per layer, and learning rate were tuned. These are crucial for the model to effectively learn complex data patterns.
5. Naive Bayes: The smoothing parameter (alpha) was tuned to address sparse data and avoid overfitting.
6. Support Vector Machine (SVM): Tuning focused on the kernel type, regularization parameter (C), and kernel coefficient (gamma) to enable SVM to find the optimal separating hyperplane.

**Figure 1:**
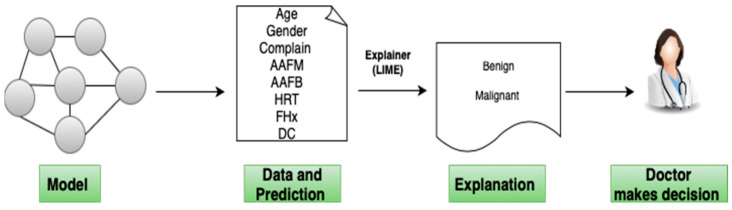
Explanation of individual predictions. A model predicts the patient’s type of cancer if none, and LIME highlights the symptoms in the patient’s history that led to the prediction. The set of risk factors contributing to the “Cancer” prediction. With this approach, a doctor can make an informed decision about whether to trust the model’s prediction.

Our variables were converted to numerical values for modeling purposes. We used Python 3.11.0 for the analysis and default parameters, except for model evaluation (explained later). Figure 1 depicts the proposed model.

In addition to machine learning, XAI techniques like LIME and SHAP were used to understand the model’s predictions. These methods helped us grasp how models make predictions and identify the most influential features. LIME and SHAP also assisted in identifying potential model issues, improving the performance, and gaining insights into the underlying data. Overall, our approach explored how machine learning can predict diagnoses while providing a deeper understanding of the models’ inner workings.

#### 3.2.1. Model Performance and Hyperparameter Tuning

Cross-validation, a common machine learning technique, was employed for both the model performance assessment and hyperparameter tuning. In this study, we utilized a 5-fold cross-validation strategy for both purposes.

##### Hyperparameter Exploration

Optimizing the model’s performance often hinges on the meticulous selection of hyperparameter values. We explored various configurations for each machine learning model:

- Specific Hyperparameters: Tuning focused on parameters specific to each model’s architecture. For instance, the number of trees and their maximum depth were explored for Random Forest, while the kernel type and cost parameter (C) were investigated for Support Vector Machines (SVMs).
- Tuning Method: Due to our dataset size, a random search approach was employed to efficiently evaluate a range of hyperparameter values. Random search samples hyperparameter values randomly from a defined range, enabling the exploration of a significant portion of the hyperparameter space while being computationally efficient for smaller datasets.
- Key Observations: Valuable insights were gained from the exploration process. For example, the Random Forest performance improved with an increasing number of trees, up to a certain point. In contrast, SVM achieved better accuracy with a linear kernel compared to a non-linear kernel for this specific dataset. These findings informed the final selection of hyperparameters used in our final models.

##### Model Evaluation Metrics

To evaluate our model’s accuracy, we utilized an accuracy classification score (Equation (1)), defined as the proportion of correctly predicted instances to the total number of instances in the dataset, expressed as a percentage. Our target variable, Bi-RADS, was used to classify each case based on the provided features.

We employed additional metrics to gain a more comprehensive understanding of the models’ strengths and weaknesses:

- Precision (Equation (2)) focuses on positive predictions, representing the ratio of true positives to the total number of elements labeled as positive.
- Recall (Equation (3)) focuses on completeness, capturing the model’s ability to identify all positive cases. It represents the ratio of true positives to the total number of elements that belong to the positive class.
- F1-Measure (Equation (4)) combines precision and recall into a single score, addressing the limitations of using just one metric.

(1) $$Accuracy=\frac{t_{p}+t_{n}}{t_{p}+t_{n}+f_{p}+f_{n}}$$

(2) $$Percision=\frac{t_{p}}{t_{p}+f_{p}}$$

(3) $$Recall=\frac{t_{p}}{t_{p}+f_{n}}$$

(4) $$F1=2*\frac{\left(precision*recall\right)}{\left(precision+recall\right)}$$

## 4. Results

In this study, we aimed to develop XAI models for predicting benign and malignant breast cancer diagnoses. The dataset consisted of the diverse clinical and pathological features of breast cancer patients, and we trained six different models using this data. The performance of these models was evaluated using widely used metrics such as accuracy, precision, recall, F1 score, and AUC-ROC score. The evaluation results of these models are presented in Table 3, showing the performance of the algorithms using the specified metrics.

**Figure 2:**
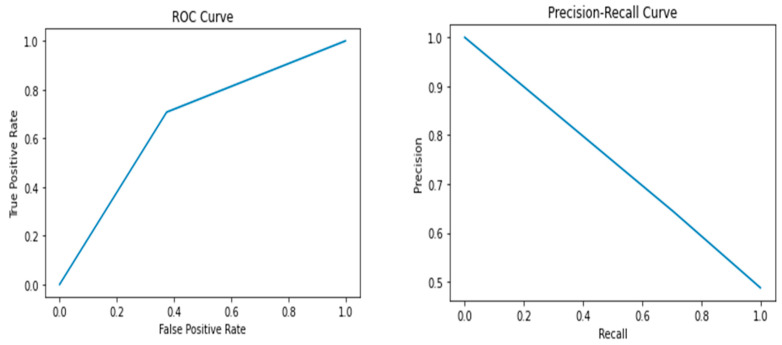
Performance metrics including precision, recall, F1 score, and AUC-ROC score for Logistic Regression.

**Figure 3:**
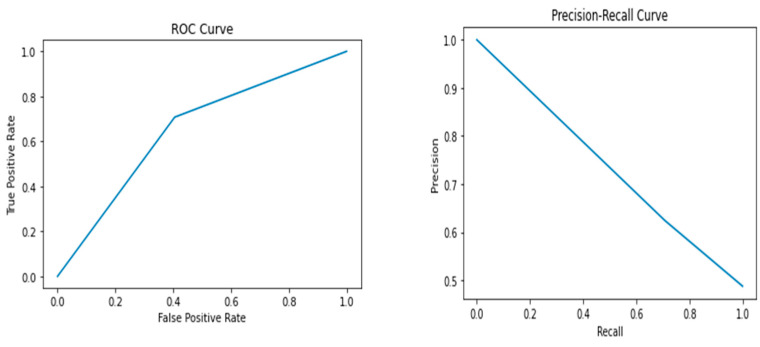
Performance metrics including precision, recall, F1 score, and AUC-ROC score for Decision Tree.

**Figure 4:**
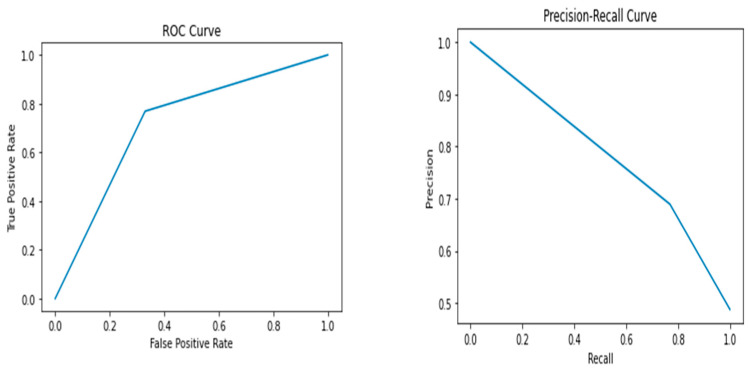
Performance metrics including precision, recall, F1 score, and AUC-ROC score for Random Forest.

**Figure 5:**
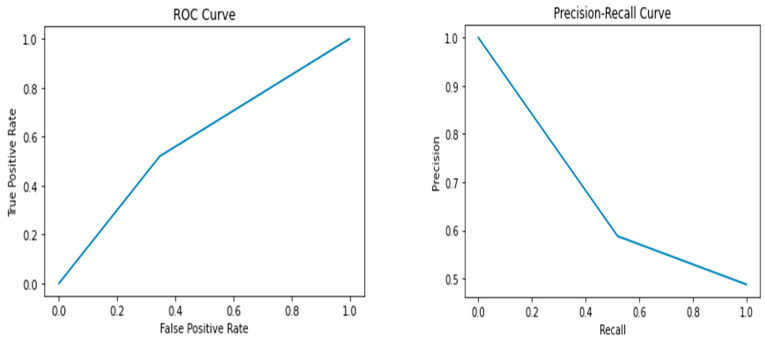
Performance metrics including precision, recall, F1 score, and AUC-ROC score for Support Vector Machine.

**Figure 6:**
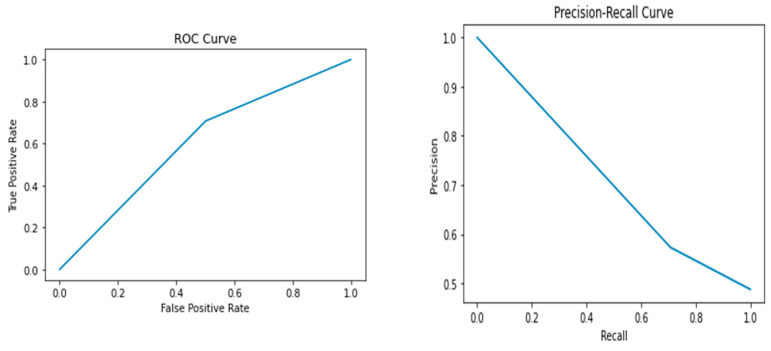
Performance metrics including precision, recall, F1 score, and AUC-ROC score for Naïve Base.

The results of the study showed that the Random Forest model performed the best, with an accuracy of 0.72, precision of 0.69, recall of 0.77, F1 score of 0.73, and AUC-ROC score of 0.72. The Random Forest model had the highest performance metrics among all the models, indicating that it can accurately predict whether a breast cancer diagnosis is benign or malignant. In contrast, the Support Vector Machine model had the lowest performance among all the models, with an accuracy of 0.59, precision of 0.59, recall of 0.52, F1 score of 0.55, and AUC-ROC score of 0.59. The Logistic Regression, Decision Tree, K-Nearest Neighbor, and Naive Bayes models had lower performance metrics than the Random Forest model, with values ranging from 0.60 to 0.67. Figure 2, Figure 3, Figure 4, Figure 5 and Figure 6 depict the performance metrics for these ML models.

The ROC curves presented in Figure 2, Figure 3, Figure 4, Figure 5 and Figure 6 serve as important visualizations for assessing the discriminative capabilities of our machine learning models. While these curves provide valuable insights into the models’ performance, it is important to address the specific observation regarding the limited number of data points.

### 4.1. RQ1: Do the Proposed ML Models Generate Consistent Lists of the Most Important Features? In Other Words, Do LIME and SHAP Yield Similar Results in Terms of Explainability?

To enhance the trust and transparency of our proposed models, we utilized LIME, an XAI approach. Initially, our analysis revealed that the RF model achieved the highest accuracy, at 72%, while Support Vector Machine had the lowest accuracy, at 59%, as presented in Table 3. However, understanding why these models performed at these levels and what factors contributed to their accuracy is a complex task that can be challenging for humans [1]. To address this issue, we employed a black box explainer, which provided critical insights into the prediction models and extracted the essential features influencing their accuracy to enhance the interpretability of our models.

**Figure 7:**
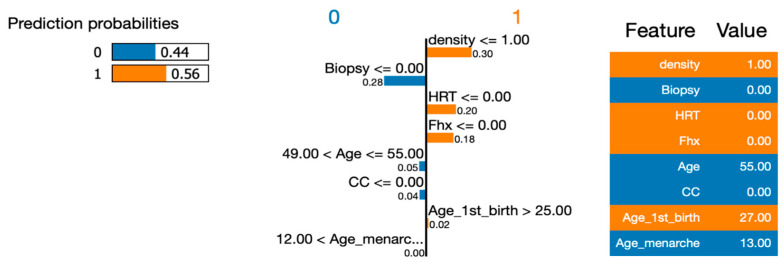
Visualizing the importance of individual features in the decision-making process of a Logistic Regression model using LIME.

**Figure 8:**
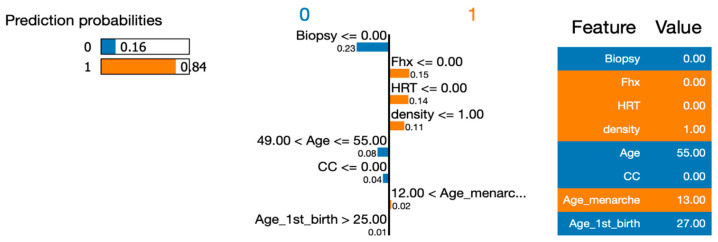
Visualizing the importance of individual features in the decision-making process of a Random Forest Classifier using LIME.

**Figure 9:**
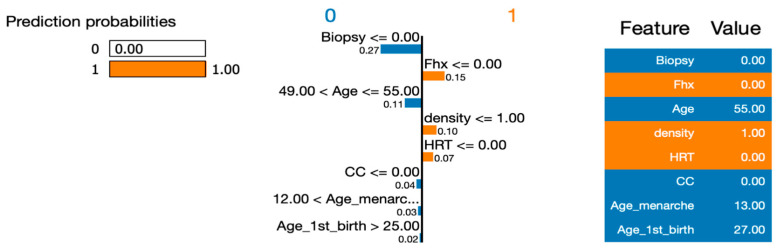
Visualizing the importance of individual features in the decision-making process of Decision Tree using LIME.

**Figure 10:**
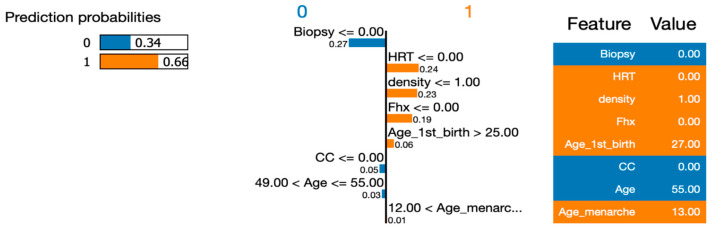
Visualizing the importance of individual features in the decision-making process of Neural Network using LIME.

**Figure 11:**
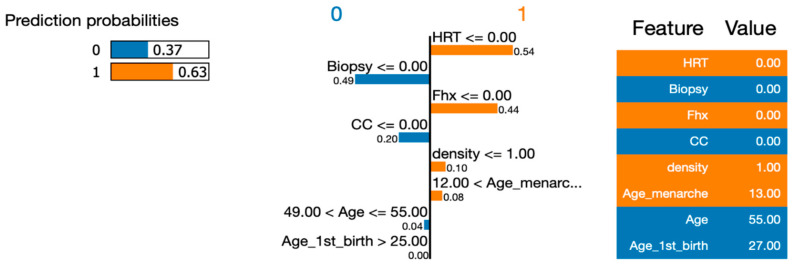
Visualizing the importance of individual features in the decision-making process of Naïve Base using LIME.

To address RQ1, we employed the LIME (Local Interpretable Model-Agnostic Explanations) approach to explain the six machine learning (ML) models and calculate the importance of their features. In order to gain deeper insights using LIME, we selected a single instance (Instance 50) at random from our dataset to be explained by all the models. By utilizing LIME, we were able to interpret and compare the results generated by the six ML algorithms. Figure 7, Figure 8, Figure 9, Figure 10 and Figure 11 visualize the importance of individual features in each ML model using LIME.

The LIME figures show the contribution of each feature to the model’s prediction for a specific test instance, which is indicated by the values in the “Value” column. The feature values are scaled to have a mean of 0 and a standard deviation of 1, and the contribution of each class (Benign or Malignant) to the prediction is indicated by the blue of the bar, where blue indicates Benign, and orange indicates Malignant.

Table 4 presents a comprehensive list of the features and their respective importance when predicting whether a given instance is classified as Benign or Malignant for each utilized model. Overall, the results indicate some similarities among the eight features in the models. However, there are notable differences in the importance rankings across the proposed models. Biopsy was identified as the primary feature (the most important feature) for Random Forest, Decision Tree and Neural Network, while density was the most important feature for Logistic Regression and Support Vector Machine. Overall, the order of features was different for every model. When we look at the top features for explainability for all six models, we see significant differences across the models. For instance, while biopsy was one of the top features for most of the models, this was not the case for SVM. As a summary, the features that were listed as the top three were, generally, biopsy (5 times), HRT (5 times), FHX (3 times), density (3 times) and age (2 times). When analyzing the models’ behavior as we progress down the list, we can observe that while there are some differences in the top four feature rankings, the models tend to simplify their assessment of features as we move towards the end. This trend becomes evident in positions 6, 7, and 8, where the least important features tend to be shared among the models.

Figure 7 shows the results obtained from the LIME analysis when explaining the logistic regression. LIME explained the probability by comparing the actual class to the targeted class values. Based on the LIME explanation plot and the associated prediction probabilities, it appears that the model predicted the instance to be malignant with a probability of 0.56. The LIME plot provides some insight into the features that contributed most to this prediction, with density being the most important feature, with a value of 1.0. However, it is important to note that the LIME explanation should be interpreted with caution considering the context of the model. Overall, the model’s performance for this instance seems to be relatively uncertain, with a predicted probability of only 0.56. A further investigation of the specific features that contributed to this prediction could potentially improve the model’s performance.

Figure 8 shows the LIME explanation plot, which provides an explanation for the decision made by the Random Forest Classification model for the given test instance. It highlights the most important features that influenced the model’s decision, and the direction of their impact on the prediction. For the Benign class, the features that contributed the most to the prediction were biopsy, age, CC, and Age_1st_birth. For example, the model predicted a higher probability of being benign when biopsy was less than or equal to 0.00, age was between 49.00 and 55.00, CC was less than or equal to 0.00, and Age_1st_birth was greater than 25.00. For the Malignant class, the features that contributed the most to the prediction were Fhx, HRT, density, and Age_menarche. For example, the model predicted a higher probability of malignancy when the Fhx was less than or equal to 0.00, HRT was less than or equal to 0.00, density was less than or equal to 1.00, and Age_menarche was greater than 12.

**Figure 12:**
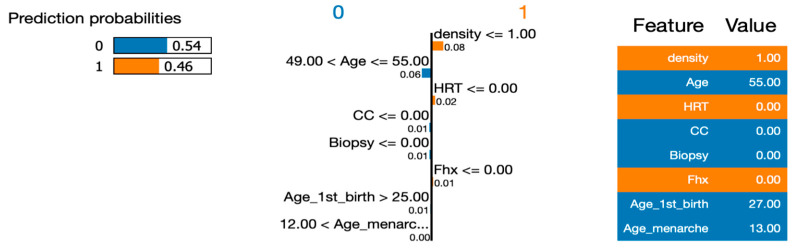
Visualizing the importance of individual features in the decision-making process of Support Vector Machine using LIME.

In Figure 9, Figure 10, Figure 11 and Figure 12, we present an insightful analysis of the Decision Tree, Neural Network, Naïve Bayes, and Support Vector Machine models. These figures provide a visual representation of how each model processes and interprets the data.

Examining the LIME (Local Interpretable Model-Agnostic Explanations) explanation plot and the associated prediction probabilities, we can gain a further understanding of the models’ predictions for a specific instance. Starting with the Decision Tree model, it confidently predicted the instance to be malignant with a probability of 1.00. This high probability suggests that the model’s decision-making process, based on the provided features, strongly supports the classification of malignancy.

Moving to the Neural Network model, we observe a lower probability of 0.66 for predicting malignancy. This indicates a relatively moderate confidence level compared to the Decision Tree model. The Neural Network might have incorporated more complex patterns and interactions among the features to arrive at its prediction. The Naïve Bayes model also predicted the instance to be malignant, albeit with a slightly lower probability of 0.63. Naïve Bayes models assume independence among the features, and although this assumption might not hold perfectly in real-world scenarios, it still yielded a reasonable prediction for this instance.

Surprisingly, the Support Vector Machine model took a different stance by predicting the instance to be benign, with a probability of 0.54. This model’s decision boundary might be influenced by different feature combinations, leading to a divergence from the other models’ predictions. Overall, these explanations provide valuable insights into the reasoning behind each model’s prediction. The Decision Tree model exhibited the highest confidence in predicting malignancy, while the Neural Network, Naïve Bayes, and Support Vector Machine models demonstrated varying degrees of confidence and divergent predictions. Such information can be instrumental in understanding the strengths, weaknesses, and interpretability of these machine learning models in the context of the given dataset.

### 4.2. RQ2: Which Features Have the Most Significant Impact on the Model’s Decision-Making Process?

**Figure 13:**
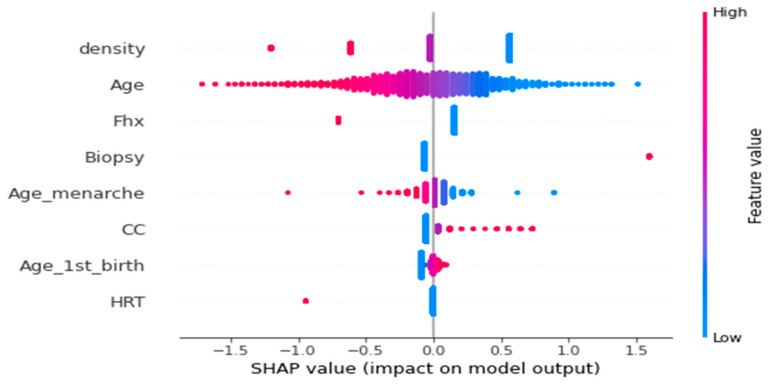
Comprehensive analysis of SHAP values for the logistic regression influencing model decisions.

To address RQ2, we employed Logistic Regression, as suggested by previous work [51], and utilized SHAP to present a summary plot for a single instance. Figure 13 displays a combined visualization of the feature importance and effects, offering insights into the model’s behavior. The y-axis ranks the features based on their importance, while each point on the summary plot represents a Shapley value for a specific feature and data point, positioned according to its Shapley value. The color of each point represents the corresponding feature value on a low-to-high scale. Overlapping points in the vertical direction depict the distribution of Shapley values for each feature. By examining the summary plots for each class, we can discern the relationship between feature values and their impact on predictions for each class. This comprehensive understanding of the model’s behavior provides valuable insights at a global level. Moreover, when analyzed per failure class, it enables the extraction of localized insights on behavior patterns.

The results indicate that density, age, and Fhx are the top three factors influencing the model’s behavior and decision making. Density and Fhx exhibit varying amounts of contributing values to the model’s decision. Notably, age and Age_menarche demonstrate the highest number of contributing values. This information can be leveraged by domain experts to either confirm or challenge the model’s behavior, enabling them to make informed decisions about placing trust in the model’s predictions.

**Figure 14:**
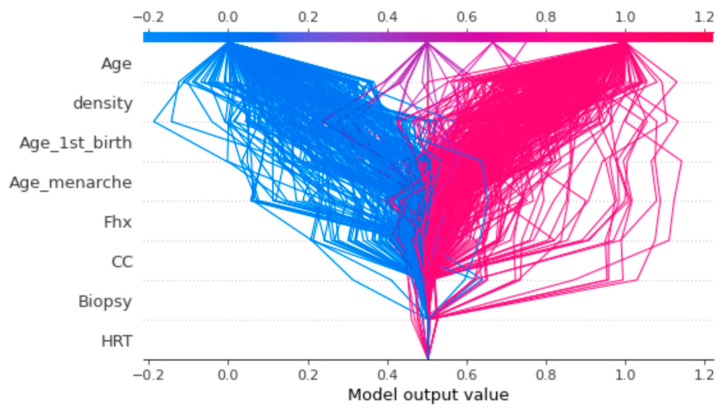
Mapping cumulative SHAP values for each prediction.

The decision plot in Figure 14 provides an insightful representation that offers a comprehensive view of the models’ decisions in the context of cumulative SHAP values for feature predications. Through a unique mapping technique, this plot captures the intricate interplay between input features and their respective contributions to prediction outcomes. As the cumulative SHAP values accumulate, the decision plot offers an intuitive understanding of how varying feature combinations influence the model’s decision-making process. This visualization not only enhances interpretability, but also serves as a valuable tool for identifying the key drivers behind specific predictions, enabling practitioners and researchers to gain deeper insights into the model’s behavior and decision rationale.

### 4.3. RQ3: What Obstacles Are Encountered in the Context of Our Study When Implementing eXplainable Artificial Intelligence (XAI)?

Assessing the effectiveness and utility of these explanations is a complex task that requires appropriate evaluation metrics and criteria to be defined. Furthermore, striking a balance between the explanation accuracy and simplicity poses a challenge. While complex models may provide accurate predictions, the explanations can be difficult for end users without technical expertise. Another significant challenge arises from the compatibility issues that can arise when different models attempt to explain a single instance. In the context of this research, it was observed that machine learning models exhibited discrepancies in their explanations for the same instance. This discordancy highlights the need for a further investigation of the underlying reasons behind such inconsistencies. To address this challenge, future research should explore the factors contributing to the discrepancies among models when explaining a single instance. This may involve analyzing the differences in model architectures, training data, or decision boundaries [52]. Additionally, it is important to assess these discrepancies’ impact on the models’ interpretability and trustworthiness. Understanding and addressing the compatibility issues among models when explaining individual instances is crucial for enhancing the reliability and applicability of eXplainable Artificial Intelligence (XAI). By identifying the sources of discrepancies and developing techniques to mitigate them, we can improve the consistency and coherence of model explanations. This will ultimately contribute to the broader goal of building transparent and trustworthy AI systems.

## 5. Discrepancies in Explanation Methods across Algorithmic Models: Analysis and Proposed Solutions

In this section, we delve into the intriguing discrepancies uncovered when applying explanation methods, specifically LIME and SHAP, across various machine learning algorithms. We explore the underlying causes of these inconsistencies and offer potential solutions to address them within the context of the algorithms under consideration. The discrepancies observed in the outcomes of the LIME and SHAP explanations when applied to different machine learning models can be attributed to several factors:

1. Algorithmic Complexity: The diverse nature of algorithms, from the simplicity of logistic regression to the complexity of neural networks, can influence how these methods interpret their output [53].
2. Feature Importance Sensitivity: Certain algorithms might assign varying degrees of importance to different features. This can lead to fluctuations in the explanation outcomes, as LIME and SHAP might respond differently to these varying importance levels [54].
3. Interactions and Nonlinearity: The complex interactions between features in algorithms like decision trees and neural networks can complicate the way LIME and SHAP interpret their behavior. Nonlinearities and interactions may not align with the assumptions these methods make [55].

### 5.1. Proposed Solutions

To address the noted discrepancies and foster more reliable explanations, we suggest the following strategies, tailored to the specific algorithms under analysis:

1. Algorithm-Specific Surrogate Models: Constructing surrogate models that mimic the behavior of each algorithm could lead to more accurate explanations. These models can be designed to capture the nuances of algorithmic interactions, potentially improving the alignment with LIME and SHAP explanations [56].
2. Model-Agnostic Ensembles: Employing ensemble techniques that combine LIME and SHAP explanations across various algorithms can provide a more holistic view. By averaging or weighting the explanations, the influence of algorithmic idiosyncrasies can be minimized [57].
3. Feature Engineering and Selection: Prioritizing feature engineering and selection tailored to each algorithm’s behavior might mitigate discrepancies. Focusing on features with a high impact within the context of each algorithm can help align the LIME and SHAP explanations.

### 5.2. Unveiling the Challenges and Opportunities in eXplainable Artificial Intelligence (XAI)

Explainable Artificial Intelligence (XAI) unfolds a spectrum of challenges and opportunities that shape its role in the realm of AI. On the one hand, XAI encounters formidable challenges that require careful consideration and innovative solutions [57]. On the other hand, it presents significant opportunities to enhance trust, transparency, and the interpretability of machine learning models. By delving into the challenges and embracing the opportunities, we can unlock the full potential of XAI in various domains.

In terms of challenges, XAI confronts complexities in several key areas. Firstly, the evaluation of the explanations provided by machine learning models poses a formidable task [52]. Assessing the effectiveness and utility of these explanations demands the definition of appropriate evaluation metrics and criteria to measure their quality and comprehensibility. Striking a balance between the explanation accuracy and simplicity also emerges as a challenge [58]. While complex models may yield accurate predictions, the explanations they generate can be difficult for non-technical end users to understand. Additionally, the compatibility issues arising when different models attempt to explain a single instance introduce complexities that warrant further investigation [50]. Furthermore, in the design of machine learning algorithms, the process of feature selection, whereby a subset of relevant features is chosen for model inputs, holds significant importance. However, it is acknowledged that feature selection can introduce nuances in the subsequent interpretation outcomes, particularly when employing methods like LIME and SHAP. To address the impact of feature selection discrepancies on the interpretation outcomes, several strategies can be considered:

1. Sensitivity Analysis: Conduct sensitivity analyses by varying the feature subsets used for different machine learning models. This allows for a comprehensive exploration of how explanations change with varying input spaces.
2. Ensemble Explanations: Employ ensemble explanation techniques that consolidate the explanations generated by models with different feature sets. By aggregating diverse explanations, these ensemble methods can provide a more comprehensive view of the model behavior across varying input spaces.
3. Consistency Measures: Develop measures to quantify the consistency of explanation outcomes when feature selection varies. This can aid in identifying the degree of stability in feature importance rankings and contributions.

### 5.3. Receiver Operating Characteristic (ROC) and Precision-Recall (PR) Analysis

The ROC curves, prominently featured in Figure 2, Figure 3, Figure 4, Figure 5 and Figure 6, offer a visualization of the models’ ability to discriminate between true positives and false positives across varying threshold values. A notable observation pertains to the inclusion of three data points within these curves. We made this strategic choice to present a succinct portrayal of the overall performance trend.

While the limited data points might not encapsulate every nuanced performance aspect, they convey a directional understanding of the models’ performance across threshold settings. By maintaining the original graph structure, we preserve the high-level insights these curves offer. The ROC curves provide valuable insights into the models’ overall discriminative capabilities, and our interpretations underscore that the depicted trends are indicative of broader performance dynamics.

The Precision–Recall (PR) curves, which are also prominently displayed in Figure 2, Figure 3, Figure 4, Figure 5 and Figure 6, offer a critical lens through which we can assess the performance of our machine learning models. In particular, PR curves hold special relevance when dealing with imbalanced class distributions, a common scenario in real-world applications. A noteworthy observation emerges from the AUPRC values, some of which are equal to or less than 0.5, signaling a nuanced aspect of model behavior.

An AUPRC value below 0.5 prompts inquiries into the models’ sensitivity and their effectiveness in recognizing rare positive instances amidst a sea of negative ones. This observation emphasizes the challenges that arise when handling imbalanced data, where correctly identifying positive instances becomes even more crucial.

These issues manifest as discrepancies among the explanations provided by different models, highlighting the need to unravel the underlying reasons behind such inconsistencies [59].

Amidst these challenges, XAI offers compelling opportunities. The enhanced trust and acceptance of machine learning models can be achieved by providing understandable explanations that shed light on the decision-making process. This empowers users to gain insights into the rationale behind AI-generated decisions and engenders confidence in the model’s predictions [60]. Furthermore, XAI facilitates collaborative decision making by fostering effective interaction between humans and AI systems [61]. Explanations enable users to comprehend the reasoning behind AI-generated decisions, enabling them to make more informed judgments based on a combination of human expertise and model insights.

Another significant opportunity lies in XAI’s potential to identify biases and errors in machine learning models. Through the analysis of explanations, researchers can uncover instances where models exhibit biased behavior or make incorrect predictions [62]. This discovery process paves the way for model improvement and fairness, ensuring the ethical deployment of AI technologies. Moreover, XAI techniques enable researchers to gain deeper insights into the relationships and patterns embedded within the data. The exploration of model explanations uncovers hidden insights, identifies critical features, and guides further research, propelling knowledge discovery in the domain.

Furthermore, XAI plays a vital role in meeting regulations and addressing ethical considerations. The transparency and interpretability of XAI models ensure adherence to regulations such as the General Data Protection Regulation (GDPR), fostering the responsible and accountable deployment of AI in sensitive domains [63,64]. Addressing these challenges through innovative techniques and methodologies will pave the way for transparent, trustworthy, and interpretable machine learning models. Ultimately, this will promote the widespread adoption of XAI, benefiting diverse domains and stakeholders alike.

### 5.4. Potential Reasons for Model Performance

Our experiments revealed that Random Forest emerged as the best-performing model for predicting Bi-RADS scores in this study, while SVM exhibited lower performance. Here are some possible explanations:

- Random Forest Strength:

Handling Complexities: Random Forests are ensemble methods that combine multiple decision trees [53]. This ensemble approach can effectively capture non-linear relationships within the data, potentially a strength for Bi-RADS prediction where various factors contribute to the score.

- Robustness to Overfitting:

Random Forest’s inherent bagging technique (randomly selecting subsets of features for each tree) helps prevent overfitting [65], a common challenge in machine learning, particularly when dealing with complex datasets.

- Potential Challenges with SVM:

Data Separability: SVMs excel at finding the optimal hyperplane to separate data points belonging to different classes [66]. However, if the data are not linearly separable in the feature space, the SVM performance might suffer. Depending on the characteristics of your dataset, the Bi-RADS scores might not be perfectly separated using linear boundaries.

Kernel Selection: SVM performance heavily relies on the chosen kernel function [65]. While we explored different kernels, it is possible that a more suitable kernel type (beyond the ones investigated) could improve SVM’s performance for this specific dataset.

## 6. Conclusions

Our research demonstrates the application of eXplainable Artificial Intelligence (XAI) in predicting the risk factors associated with breast cancer in the Saudi population. Through the evaluation of various classifiers, the Random Forest model emerged as the top performer, exhibiting high accuracy, precision, recall, F1 score, and AUC-ROC score values. This indicates its effectiveness in accurately distinguishing between benign and malignant breast cancer diagnoses.

The utilization of a black box explainer yielded valuable insights into the prediction models, uncovering the most influential features that contribute to their accuracy. These insights have the potential to enhance decision making in breast cancer diagnosis and treatment, enabling medical professionals to prioritize relevant factors and customize interventions accordingly. Additionally, the validation analysis of features ensures alignment with existing medical knowledge, reinforcing trust in the model’s predictions. The transparency and interpretability offered by XAI play a crucial role in fostering trust in the model’s predictions, empowering medical professionals to make well-informed assessments of its reliability. Furthermore, identifying influential features opens avenues for further research, providing deeper insights into the underlying biological or clinical mechanisms related to breast cancer. This has the potential to drive advancements in knowledge, uncover novel biomarkers, and enhance our understanding of risk factors. In summary, our study highlights the practical implementation of XAI in improving decision making, bolstering model trust and interpretability, and generating valuable research insights in the field of breast cancer. By harnessing the capabilities of XAI, we can contribute to more accurate diagnoses, personalized treatment strategies, and advancements in breast cancer research, ultimately leading to improved patient outcomes.

## Figures and Tables

**Table 1:** Summary of major studies assessing the impact of ML techniques on breast cancer diagnosis.

Authors	Year	Dataset	Algorithms	Variables/Inputs	Results
Agarap [10]	2018	Wisconsin Diagnostic Breast Cancer (WDBC)	GRU-SVM, Linear Regression (LR), Multilayer Perceptron (MLP), Nearest Neighbor (NN) search, Softmax Regression, and Support Vector Machine (SVM)	Digitized images of a fine needle aspirate (FNA) tests on a breast mass	MLP algorithm stands out among the implemented algorithms with a test accuracy of ≈99.04%.
Amrane et al. [11]	2018	Unspecified	Naive Bayes (NB) classifier and k-nearest neighbor (KNN)	Breast cancer symptoms (constant pain, changes in the size, color (redness), skin texture of breasts)	KNN gave the highest accuracy (97.51%) with the lowest error rate compared to the NB classifier (96.19%).
Sherafatian [12]	2018	miRNA-seq dataset	Tree-based algorithms	Tumors and symptoms	Classified three types of tumors that can be used as biomarkers
Tseng et al. [13]	2019	Clinical dataset	Random Forest (RF), SVM, LR, Bayesian Classification (BC)	Serum biomarkers and clinicopathological data	RF-based model was determined to be the optimal model to predict breast cancer metastasis at least 3 months in advance
Ferroni et al. [14]	2019	Clinical dataset	ML-Decision Support System with Random Optimization	Demographic, clinical and biochemical data	the model was capable of stratifying the testing set into two groups of patients with low- or high-risk of progression
Omondiagbe et al. [15]	2019	WDBC	SVM, ANN & NB	Tumors and symptoms	A hybrid approach to breast cancer diagnosis that involves reducing the high dimensionality of features using linear discriminant analysis (LDA), and then applying the new reduced feature dataset to SVM, was adopted, resulting in an accuracy of 98.82%, sensitivity of 98.41%, and specificity of 99.07%.
Tapak et al. [16]	2019	Clinical dataset	NB, RF, SVM, LR	Various factors in EHRs of breast cancer patients	In the prediction of survival, the average specificity of all techniques was ≥94%, and the SVM and LDA have greater sensitivity (73%) in comparison to other techniques.
Mojrian [17]	2020	WDBC	Extreme learning machine (ELM) classification model integrated with radial basis function (RBF) kernel called ELM-RBF, SVM	Symptoms	ELM-RBF outperformed the linear SVM model, with an RMSE, R 2 and MAPE equal to 0.1719, 0.9374 and 0.0539, respectively
Chaurasia & Pal [18]	2020	WDBC	LR, DT, SVC, KNN, RF, NB	Symptoms	All ML algorithms perform best, with test accuracy exceeding 90%
Turkki et al. [19]	2019	Clinical dataset	DRC classifier	tissue microarray (TMA) samples	The accuracy (C-index) of the DRS grouping was 0.60 (95% CI 0.55–0.65), compared to 0.58 (95% CI 0.53–0.63) for human expert predictions based on the same TMA samples.
Abdar and Makarenkov [20]	2019	WDBC	SVM, ANN, confidence-weighted voting method and the boosting ensemble technique (CWV-BANNSVM)	Symptoms	CWV-BANNSVM model was able to improve the performance of the traditional machine learning algorithms applied to BC detection, reaching an accuracy of 100%.
Abdar et al. [21]	2020	WDBC	NB		SV-BayesNet-3-MetaClassifier and SV-Naïve Bayes-3-MetaClassifier achieved an accuracy of 98.07% (K = 10)
Singh [22]	2019	Clinical dataset	K-NN, SVM, NB		K-NN classifier achieves the highest classification accuracy, at 92.105%, followed by medium Gaussian SVM, which achieves a classification accuracy of 83.684%
Liu et al. [23]	2018	Clinical dataset	SVM, RF, DT, Adaboost		Accuracy of RF is higher than the other three methods
Khourdifi and Bahaj [24]	2018	Clinical dataset	RF, NB, SVM, K-NN		SVM gives the highest accuracy, at 97.9%

**Table 2:** Description of extracted features.

Variable	Types of Variables	Description	
Age	Age years	Age at breast cancer diagnosis or screening	
Gender	Categorical variable	Male (0), Female (1)	
Complain (CC)	Categorical variable	0 = Screening 1 = Pain 2 = Lump 3 = Scar 4 = Moles 5 = Skin retracting	6 = Tissue thickening 7 = Nipple discharge 8 = Nipple inversion 9. Other
Age Menarche (AM)	Discrete variable	Age at first menstruation	
Age at first birth (AB)	Discrete variable	Age of women at birth of first child	
Hormone Replacement Therapy (HRT)	Categorical variable	Indicates whether the patient has undergone any hormone replacement therapy.	
Family Health History (FHx)	Categorical variable	History of breast cancer in a first-degree relative	
Density Class (DC)	Categorical variable	1 = Almost entirely fat 2 = Scattered fibro glandular densities 3 = Heterogeneously dense 4 = Extremely dense 5 = Unknown or different measurement system	
BI-RADS	Breast imaging reporting and data system	1: Benign 2: Malignant	

**Table 3:** Machine learning models’ performance.

Algorithm	Accuracy	Precision	Recall	F1 Score	AUC-ROC
Random Forest (RF)	0.72	0.69	0.77	0.73	0.72
Logistic Regression (LR)	0.67	0.64	0.71	0.67	0.67
Decision Trees (DT)	0.65	0.62	0.71	0.66	0.65
Neural Network (NN)	0.63	0.58	0.82	0.68	0.63
Naïve Bayes (NB)	0.60	0.57	0.71	0.63	0.60
Support Vector Machine (SVM)	0.59	0.59	0.52	0.55	0.59

**Table 4:** Ordered list of features ranked by importance, as determined by LIME. Features in bold or underlined indicate consistency across the models’ lists.

Feature Rank	1	2	3	4	5	6	7	8
RF	**Biopsy**	**Fhx**	**HRT**	**Density**	**Age**	**Complain**	**AM**	**AB**
LR	Density	Biopsy	**HRT**	Fhx	**Age**	**Complain**	AB	AM
DT	**Biopsy**	**Fhx**	Age	**Density**	HRT	**Complain**	**AM**	**AB**
NN	**Biopsy**	HRT	Density	Fhx	AB	**Complain**	Age	AM
NB	HRT	Biopsy	Fhx	Complain	Density	AM	Age	**AB**
SVM	Density	Age	**HRT**	Complain	Biopsy	Fhx	AB	AM

## Data Availability

The data presented in this study are available upon request from the corresponding author. The data is not publicly available since it was obtained after the approval from Institutional Review Board as mentioned above.
